# Health Services, Socioeconomic Indicators, and Primary Care Coverage in Mortality by Lower Genital Tract and Breast Neoplasias in Brazilian Women during Reproductive and Non-Reproductive Periods

**DOI:** 10.3390/ijerph17165804

**Published:** 2020-08-11

**Authors:** José Lucas Souza Ramos, Francisco Winter dos Santos Figueiredo, Lea Tami Suzuki Zuchelo, Flávia Abranches Corsetti Purcino, Fernando Adami, Rodrigo Goncalves, Carlos Alberto Ruiz, Edmund Chada Baracat, José Maria Soares Junior, Isabel Cristina Esposito Sorpreso

**Affiliations:** 1 Disciplina de Ginecologia, Departamento de Obstetrícia e Ginecologia, Faculdade de Medicina, Universidade de São Paulo, São Paulo, SP 05403-000, Brazil; joselucas@usp.br (J.L.S.R.); leasuzuki@usp.br (L.T.S.Z.); flavia.purcino@hc.fm.usp.br (F.A.C.P.); caruiz@terra.com.br (C.A.R.); ecbaracat@usp.br (E.C.B.); jose.msjunior@fm.usp.br (J.M.S.J.); 2 Epidemiology and Data Analysis Laboratory, Faculdade de Medicina do ABC (FMABC), Santo André, SP 09060-870, Brazil; francisco.figueiredo@fmabc.br (F.W.d.S.F.); adamifernando@uol.com.br (F.A.); 3 Setor de Mastologia, Disciplina de Ginecologia, Hospital das Clínicas, Faculdade de Medicina, Universidade de São Paulo, São Paulo, SP 01246-000, Brazil; rodrigo.g@hc.fm.usp.br

**Keywords:** neoplasms, women’s health, primary care coverage

## Abstract

Women’s health assistance at the low-complexity level is focused on the most common diseases and can be affected by primary health care coverage, particularly in areas far away from large urban centers. Thus, in this work, we aim to analyze the relationship between socioeconomic status, health care indicators, and primary care coverage in mortality from neoplasms of the lower genital tract and breast in Brazilian women during reproductive and non-reproductive periods. We conducted an ecological study at the Gynecology Discipline, Medicine School, University of São Paulo. Secondary data were collected from women according to reproductive periods and mortality data from the Mortality Information System based on International Classification of Disease—10th edition regarding breast and lower genital tract neoplasms in 2017. The health service and socioeconomic indicators were obtained from the Informatics Department of the Unified Health System and Brazilian Institute of Geography and Statistics. Our results showed that primary care coverage and health service indicators were not associated with mortality from breast cancer and the female lower genital tract, both in reproductive and non-reproductive periods. Sociodemographic indicators were found to be associated with mortality from breast cancer and the female lower genital tract, with income being associated with reproductive period (*β* = −0.4; 95% CI, −0.8 to −0.03) and educational level in the non-reproductive period (*β* = 9.7; 95% CI, 1.5 to 18.0).

## 1. Introduction

Breast cancer is the most frequent type of cancer and the leading cause of death in women in developed and developing countries. Cervical cancer is the second most common cancer, which occurs in the female genital tract [1,2]. In Brazil, other neoplasms of the female lower genital tract are also widely experienced, such as uterine body cancer, which is currently the third most prevalent; ovarian cancer, which is now the fourth most common [3,4]; and vulvar cancer, which is the fifth most common malignancy of the female lower genital tract [4,5,6].

Brazil is a heterogeneous, developing country and has different prevalences of mortality from breast and lower genital tract cancer in its regions and unequal access to public health services, including the prevention [7] and early detection of breast and cervical cancer [8].

Assistance for women’s health, in primary health care, focuses on the most common diseases [7] and employs specific diagnoses during the reproductive and non-reproductive periods, with different approaches for each period. Primary health care (PHC) mainly covers areas that are very distant from large urban centers, showing disparities between Brazilian regions, and this occurs in areas with low socioeconomic and educational levels [9,10]. It is known that the risk factors for breast and lower genital tract cancer are different in the reproductive and non-reproductive periods, but the prevention and screening strategies in primary care are the same in both periods of life according to our policies and public reforms [11], even for regions with a high mortality and low socioeconomic status.

Thus, our aim in this work is to evaluate the current situation and the possible relationship between primary care coverage and breast and female lower genital tract cancers, considering the periods of reproductive and non-reproductive life, health services, and socioeconomic indicators in the different federal units of Brazil.

## 2. Materials and Methods

### 2.1. Study Design

This ecological study was carried out at the Gynecology Discipline, Department of Obstetrics and Gynecology, Medical School of the University of São Paulo, on the coverage of primary care for lower genital tract and breast cancer in women, with data referring to the year 2017.

### 2.2. Data Source and Collection Procedure

We analyzed the 26 Brazilian federative units, divided in five regions (north, northeast, southeast, south, and midwest) with distinct climatic and socioeconomic characteristics. The population data are from the Brazilian Institute of Geography and Statistics (IBGE—www.ibge.gov) [12].

Mortality data were extracted from the Mortality Information System (acronym in Portuguese: SIM), included in the Informatics Department of the Unified Health System (acronym in Portuguese: DATASUS, www.datasus.gov.br). Deaths due to lower genital tract and breast cancer are registered according to the International Classification of Disease, 10th edition (ICD-10) as follows: malignant neoplasm of breast—C50; malignant neoplasm of vulva—C51; malignant neoplasm of vagina—C52; malignant neoplasm of cervix uteri—C53; malignant neoplasm of corpus uteri—C54; malignant neoplasm of uterus, part unspecified—C55; malignant neoplasm of ovary—C56; malignant neoplasm of other and unspecified female genital organs—C57 [13].

The data referring to primary care coverage were extracted from the Primary Care Information System (acronym in Portuguese: SIAB), also included in DATASUS (www.datasus.gov.br) [14].

The health indicators (number of beds, physicians, nurses, and mammography devices per 100,000 inhabitants) were extracted from the National Register of Health Establishments (acronym in Portuguese: CNES), also included in DATASUS (www.datasus.gov.br) [15].

Finally, the socioeconomic indicators (GINI index, female illiteracy rate per 100,000 inhabitants, per capita income, and population’s average years of study) were extracted from the National Household Sample Survey (acronym in Portuguese: PNAD), from the Brazilian Institute of Geography and Statistics (IBGE—www.ibge.gov) The GINI index is a coefficient that measures inequality in terms of income distribution in each group, numerically varying from zero to one (the higher the value, the higher the inequality). Per capita income is an indicator that is obtained by dividing national income by the number of inhabitants in the place (the currency exchange rate is variable; in December 2017, 1 U.S. dollar was equivalent to 3.31 Brazilian reals) [16].

### 2.3. Inclusion Criteria

The population was divided according to age, in which the “reproductive period” included patients between 15 and 49 years old and the “non-reproductive” period included patients over 50 years old, based on the standardization of age by the direct method, according to the World Health Organization (WHO) [17]. The stratification of the reproductive and non-reproductive was is based on DATASUS and national epidemiological studies [18,19].

### 2.4. Statistical Analysis

Quantitative variables were described as measures of central tendencies according to their adherence to Gaussian distribution. Crude mortality rates were calculated by the ratio between the number of deaths reported by cancer in the lower genital tract and breast in 2017 for every 100,000 women according to age groups. Pearson’s correlation test was used to analyze the correlation between primary care coverage and mortality according to reproductive periods.

Linear regression adjusted for socioeconomic and health service indicators was used to estimate the median differences in the main causes of mortality in reproductive and non-reproductive periods. The stepwise backward strategy was used with the entry criteria of *p* < 0.20 and removal of *p* = 0.05. For all analysis, a 5% confidence level was considered. The program used was Stata (StataCorp, LC, College Station, TX, USA) version 11.0.

## 3. Results

Mortality rates in Brazilian Federal Units, grouped by administrative region, from breast and lower genital tract cancers per 100,000 women are shown in Table 1. Across Brazil, 7348 deaths were registered in the reproductive period, with a mortality rate of (per 100,000 inhabitants) 12.9 (confidence interval (CI): 11.9; 13.9); in the non-reproductive period, 24,335 deaths were registered, with a mortality rate of 90.9 (CI: 86.7; 95.2).

The highest rates occurred in the northern region, both for the female population in general (37.59) and for women in the reproductive period (13.95) compared to other regions. For the non-reproductive period, the highest mortality rate was observed in the central-west region (94.28). Regarding the federative units, the state of Amazonas stands out, with the highest mortality rate both in the general population (49.80) and in the reproductive period (19.80) and in the non-reproductive period (121.00).

The primary care coverage is also shown in Table 1, showing that the northeast region has the highest coverage (85.90%). The federal units with the lowest coverage were Distrito Federal (57.6%), São Paulo (59.6%), and Amazonas (62.7%).

Regarding the characteristics of the health and socioeconomic services of Brazilian regions and states, it was observed that the largest number of physicians per 100,000 inhabitants is found in the south and southeast regions, with an average of 1.6 for both; for nurses, the largest distribution is in the south (105.3), and the largest number of beds is in the northeast and south (1.7). As regards the rate of mammography devices, the southern region shows the largest amount, with an average of 1.6, as well as the lowest GINI index (0.47). The illiteracy rate is higher in the northeast (17.9) and the average year of study is higher in the southeast (8.7). The average per capita income is higher in the central-west region (490.42) (Table 2).

Regarding mortality rates, according to the ICD-10, breast cancer presented the highest rate in the female population in general (18.9), in the reproductive period (6.4), and in the non-reproductive period (48.6); this was followed by cervical cancer, with a mortality rate of 7.3 in the general population, 3.9 in the reproductive period, and 15.5 in the non-reproductive period (Table 3).

Adjusting for all the confounding variables, primary care coverage did not show a statistically significant relationship with mortality when stratified by women’s life periods. The adjusted model showed that, in the reproductive period, low income per capita is associated with high mortality rates (*β* = −0.4; ranging from −0.8 to −0.03; *p* = 0.032), and in the non-reproductive period, the high average number of years studied is a factor associated with the high mortality rate (*β* = 9.7; ranging from 1.5 to 18.0; *p* = 0.022) (Table 4).

**Figure 1:**
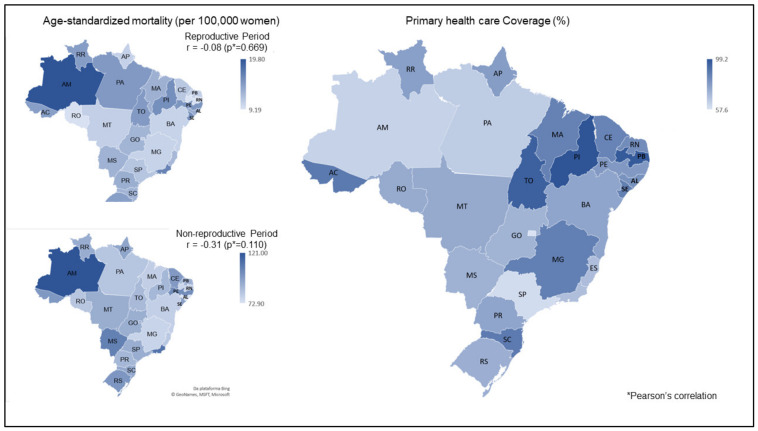
Correlation between primary care coverage and distribution of standardized mortality by age, reproductive, and non-reproductive period (per 100,000 women) and genital and breast cancer in women residing in the Federative Units and the Brazilian Federal District in 2017.

There was no significant correlation between primary care coverage and mortality from neoplasms of the lower genital tract and breast according to reproductive periods (*r* = −0.31; *p* = 0.110 for the non-reproductive period group and r = −0.08; *p* = 0.669 for the reproductive period group) (Figure 1).

## 4. Discussion

There was no correlation between primary care coverage and mortality from neoplasms of the lower genital tract and breast in women according to reproductive periods. However, per capita income was associated with an increase in mortality in women in the reproductive period and average years of study in the non-reproductive period. The PHC coverage in Brazil has increased throughout the national territories; this has been particularly true since 2010, with increases in the screening for cervical and breast cancer. However, there are still socioeconomic inequalities, which affect access to health care or delay the screening of the main causes of cancer in the most vulnerable populations, and therefore its influence on reducing mortality has not yet been significant [11].

In a study carried out in 2010 analyzing mortality rates in Brazil between 2003 and 2007, there was a higher rate of cervical cancer in the northern region while the mortality rates of breast cancer were higher in more developed regions (south and southeast), highlighting the states of Rio de Janeiro, São Paulo, and Rio Grande do Sul [20].

A study published in 2014 presenting an analysis of the period from 1980 to 2010 showed decreasing mortality from breast cancer in the capitals of the south, southeast, northeast, and midwest regions, and an increase in inland cities of the northeast and capitals of the north [21].

These studies show a change in the profile of deaths due to breast and cervical cancer. In the past, most cases occurred in large urban centers; today, the highest mortality occurs in regions with less urban flow, such as the northeast.

This increase in mortality from breast cancer may be associated with an increase in the reporting of new cases. As the increase in deaths from this neoplasm has occurred mainly in urban centers, the lifestyle of the population seems to influence the onset of cancer. The incidence of cervical and uterine cancers, on the other hand, may have decreased in large centers due to the current possibilities of prevention (Pap smear exam), and mortality rates may have been influenced by the improvement of sociodemographic indicators such as income and education [22]. However, current data from the National Cancer Institute show that breast and lower female genital tract neoplasms continued to grow in Brazil between 2010 and 2017 [23].

An important point to be highlighted in our study is that the state with the highest mortality in the northern region was Amazonas, which also has less coverage in primary care. This database was not observed in previous studies, and the state with the lowest coverage was the state of Pará. In 2016, for example, according to Neves et al. [24], both states showed the tendency of an increasing mortality rate; however, Pará had the lowest primary care coverage, with a value of 54.5%, when compared to Amazonas, with 58.4%.

The study by Garnelo et al. [25] shows a similar result: from 2013 to 2014, PHC coverage was lower in Pará and higher in Amazonas. They also analyzed coverage in the rural population and pointed out difficulties for populations that are not close to river areas. In the states of Pará and Amazonas, it is observed that family health teams are located in the tributaries of the rivers, and PHC coverage in the most distant territories is not possible, which may be one of the factors contributing to the reduction of primary care coverage.

In a publication carried out by the National Program for the Improvement of Access and Quality of Primary Care in the State of Amazonas in [26], it was observed that coverage is insufficient, especially in rural populations, where, even with the offer of preventive exams at no cost, it is not possible for residents to attends the exams due to a deficit in adequate transport [26].

Neves et al. [24] reported in a previous study that, in 2016, the southeast region had the worst PHC coverage, demonstrating an improvement in PHC coverage in the central-west region. However, in the present study with data from 2017, the midwest region still had less than adequate primary care coverage (below 70%), while the northeast region had the highest coverage of primary care, followed by the north, south, and southeast.

The coverage of primary care in a territory reflects the presence of family health teams. Thus, the greater coverage of primary care in a territory, the greater detection of cases in women and, consequently, the greater rates of reporting and mortality [27].

Regarding mortality due to breast and female lower genital tract cancer, the highest rate was shown for breast cancer, followed by cancer of the cervix, corpus uteri, ovaries, and others. These data are corroborated by the National Cancer Institute and other epidemiological studies, in which gynecological cancers, with the exception of cervical cancer, show a lower mortality and lower incidence in the population [28].

With the exception of breast and cervical neoplasms, the mortality of other gynecological neoplasms (ovary, corpus uteri, and vulva), are not significantly affected by the performance and coverage of PHC units [29]. Thus, the assistance provided by the service in the face of other neoplasms is still unviable due to the few screening strategies that are available [30,31].

Although the causality of the observed data has not been established, a systematic review by Batista and Caldas [32] showed that some points can interfere with the adherence of women over 50 to cancer prevention programs, especially difficulty in accessing health services, myths, and prejudices that are associated with sexual activities and the need to carry out complementary and gynecological exams, the poor training of health professionals to work in women’s health, and the lack of investment in women’s health education activities. These facts generate reflections both from the point of view of primary care coverage, in the face of mortality from the neoplasms presented, as well as the importance of prevention and early detection of the other (vulva, vagina, and uterine body) gynecological neoplasms.

Socioeconomic factors such as income inequality and education level are among the most important social indicators, and their impact on breast and lower genital tract mortality were analyzed in this study. It is well established that people with low incomes are at an increased risk of an array of adverse health outcomes and more likely to die prematurely [33].

Our results show that in the reproductive period, the increase in mortality was associated with lower incomes for women. In order to understand the results associated with socioeconomic and health indicators, it is necessary to analyze the economic and health particularities that exist in each Brazilian region.

Socioeconomic indicators such as the GINI index, per capita income, literacy level, and average years of schooling showed better results in the south and southeast regions, while the north and northeast regions showed more discouraging results. The south and southeast regions in particular have a peculiar characteristic: the great amount of existing urban development and the concentration of industry in these places allows the improvement of these results. The midwest region, on the other hand, presents average results, which may be associated with its urban and rural profile, concentrated in the Brazilian capital Distrito Federal and large states such as Goiás [34].

The health indicators follow the same pattern as the previous indicators, except for the variables of the number of nurses and beds in the Brazilian healthcare system, which presented approximately similar values between regions. The presence of physicians and mammography rates are variables that showed lower values in the north and northeast regions; as a consequence, higher mortality rates were also shown in these regions, for example, from breast cancer, since the difficulty in accessing a mammography exam causes a delay in diagnosis, which reduces the chances of treatment and survival [35,36,37].

Duarte and Teixeira [38] showed in a systematic review that a low socioeconomic level is associated with cervical cancer and a high socioeconomic level with breast cancer, which may indirectly explain the association of low income and mortality shown in the reproductive period.

Regions with the highest rates of social inequality and the lowest levels of human development presented the highest standardized mortality rates for cervical cancer, without a relationship with the distribution of health services offered to the population in a recent publication [39].

It is observed in the literature that neoplasms are one of the main causes of death in this period; when they do not occupy the first position, they are in second [40]. In the study by Madeiro et al. [41], on the other hand, the main group of the basic causes of death in women in reproductive period was that of neoplasms, including breast, cervix, and ovarian cancer, as shown in the research by Pitilin and Sbardelotto [42].

Regarding breast cancer, the literature demonstrates that a low socioeconomic status is associated with an increased risk of aggressive premenopausal breast cancers as well as late stage of diagnosis and poorer survival [43], corroborating the findings of this study.

This information largely impacts breast cancer care in Brazil since the country has been showing a significant upward trend for mortality from breast cancer among women between 20 and 49 years of age [44].

However, the improvement in the distribution of financial incomes and living standards in developing countries has been accompanied by an increase in breast cancer incidence and mortality in women, who have adopted new habits and living conditions, exposing themselves to a more significant number of risk factors that can trigger the disease [45,46]. It is known that several factors that influence hormonal status (e.g., age at the birth of first child) or are markers of change in hormonal status (e.g., age at menarche and age at menopause) are associated with the risk of breast cancer [47].

In the non-reproductive period, the highest average of years of study was associated with increased mortality from the studied neoplasms. This result, although confusing, can be better explained by the growth of health indicators and determinants such as socioeconomic factors in Brazil. Breast cancer, in particular, tends to show growth associated with increased life expectancy, which also follows the growth in per capita income, higher education in the population, and a decrease in the fertility rate [27].

In Brazil, there is an increasing number of nulliparous women and a low fertility rate, and there has been a postponement of pregnancy to an older age in recent decades [48]. This is due to professional investment and the development of better living conditions, which is an important association for the development of breast cancer [49] and can possibly illustrate the negative impact of increased years of study on mortality for non-reproductive women.

The importance of preventing and detecting neoplasms of the breast and the female lower genital tract at an early stage has been highlighted in different periods of the life cycle (mainly reproductive) and diagnosed in the non-reproductive period.

Furthermore, it is well known that the implementation of effective programs for cervical cancer prevention and control through the realization of regular and timely cytopathologic tests in asymptomatic women enables prevention and early oncologic diagnosis, minimizing cervical cancer mortality in the country [50], and that availability may not be possible in low-income areas.

A limitation of the study is the possible fragility of the use of secondary sources from the DATASUS and IBGE databases. However, the improvement in the completeness of epidemiological variables in cancer-related deaths over recent years must be considered, making this system an important national tool for access to mortality data and the development of ecological studies [51].

Our study is also limited by the quality of the secondary databases, as it is not possible to assess the causality of associations in the cases. However, the statistical associations found can guide the development of strategies for the prevention and control of neoplasms in primary care, especially regarding service coverage. Another limiting factor is the multifactorial nature of neoplasms, which may have different characteristics. However, studying them can have positive consequences for the analysis of PHC coverage, as well as generating conditions for the analysis of gynecological neoplasms, highlighting the preventive and multifactorial character addressed in primary care.

## 5. Conclusions

Mortality from breast and female lower genital tract neoplasms in Brazil is not associated with primary care coverage or health service indicators, both in reproductive and non-reproductive periods.

Sociodemographic indicators are associated with mortality from breast and female lower genital tract cancer, with income being associated with mortality in the reproductive period and educational level being associated with mortality in the non-reproductive period.

Health care policies for women and actions to prevent breast and lower genital tract cancer in primary health care should include intersectoral strategies that consider improvements in health determinants such as education and socioeconomic level, as well as the specifics of each period of life.

## Figures and Tables

**Table 1:** Mortality standardized by age, genital, and breast cancer according to reproductive and non-reproductive periods and primary care coverage, administrative region, and federative units in Brazilian women in 2017.

Region/ Federative Unit	Age-Standardized Mortality (per 100,000 Women)			Primary Care Coverage (%)
	Total	Reproductive Period	Non-Reproductive Period	
**North ^†^**	37.59	13.95	93.77	77.20
RO	31.00	9.36	82.60	74
AC	39.10	14.30	98.10	88.4
AM	49.80	19.80	121.00	62.7
RR	37.20	14.40	91.30	77.2
PA	34.90	14.70	82.80	64.3
AP	35.10	10.50	93.70	78.1
TO	36.00	14.60	86.90	95.7
**Northeast ^†^**	34.59	12.58	86.80	85.90
MA	32.80	12.90	80.00	85.9
PI	36.80	15.40	87.60	99.2
CE	36.80	11.20	97.50	85.3
RN	33.80	10.50	89.00	82.7
PB	29.30	9.19	77.30	97.4
PE	40.90	14.90	102.00	79.3
AL	30.90	13.20	72.90	81.1
SE	39.10	15.50	95.20	86.4
BA	30.90	10.40	79.70	75.7
**Southeast ^†^**	35.68	12.60	90.43	70.80
MG	30.40	10.40	78.00	86.7
ES	32.90	12.30	81.80	68.6
RJ	44.50	16.30	111.00	68.3
SP	34.90	11.40	90.90	59.6
**South ^†^**	36.93	13.37	92.97	78.83
PR	35.10	13.10	87.50	75.7
SC	36.40	12.70	92.70	88.1
RS	39.30	14.30	98.70	72.7
**Midwest ^†^**	36.18	11.70	94.28	68.78
MS	40.50	12.80	106.00	71.9
MT	34.40	10.70	91.00	74.2
GO	35.70	12.60	90.60	71.4
DF	34.10	10.70	89.50	57.6

^†^ Brazillian regions/RO: Rondônia/AC: Acre/AM: Amazonas/RR:Roraima/PA: Pará/AP: Amapá/TO: Tocantins/MA: Maranhão/PI: Piauí/CE: Ceará/RN: Rio Grande do Norte/PB: Paraíba/PE: Pernambuco/AL: Alagoas/SE: Sergipe/BA: Bahia/MG: Minas Gerais/ES: Espirito Santo/RJ: Rio de Janeiro/SP: São Paulo/PR: Paraná/SC: Santa Catarina/RS: Rio Grande do Sul/MS: Mato Grosso do Sul/MT: Mato Grosso/GO: Goiás/DF: Distrito Federal.

**Table 2:** Characteristics of the health and socioeconomic services of Brazilian federative units in 2017.

Region/ Federative Unit	Physicians *	Nurses *	Beds *	Mammography Device *	GINI ** Index	Illiteracy *	Average Years of Study	per Capita Income (US$)
**North ^†^**	1.0	101.7	1.6	0.9	0.54	9.1	7.9	269.24
RO	1.1	79.2	2.0	0.9	0.46	7.8	7.3	291.51
AC	1.1	106.0	1.6	0.4	0.57	14.4	7.1	234.14
AM	0.9	78.5	1.3	1.7	0.60	7.1	8.5	262.05
RR	1.4	132.6	1.8	1.0	0.55	6.7	9.0	305.47
PA	0.7	63.2	1.4	0.6	0.52	9.9	7.3	220.42
AP	0.9	95.4	1.3	0.3	0.59	6.0	8.7	284.80
TO	1.2	156.8	1.5	1.4	0.50	11.5	7.7	286.22
**Northeast ^†^**	1.1	93.3	1.7	1.3	0.55	17.9	6.9	243.60
MA	0.7	86.9	1.8	0.6	0.54	19.7	6.5	182.51
PI	1.0	99.1	2.1	1.3	0.54	20.6	6.6	229.43
CE	1.0	83.8	1.6	0.9	0.56	16.1	7.1	253.32
RN	1.1	93.2	1.7	1.1	0.53	15.7	7.2	258.85
PB	1.2	126.3	1.8	2.9	0.56	20.4	6.8	283.14
PE	1.2	96.7	1.8	1.3	0.56	14.7	7.3	263.08
AL	1.1	77.8	1.4	1.2	0.53	22.2	6.4	200.60
SE	1.3	80.5	1.1	1.3	0.56	16.8	7.1	254.71
BA	1.0	95.5	1.6	1.2	0.60	14.7	7.0	266.68
**Southeast ^†^**	1.6	99.7	1.4	1.2	0.52	4.6	8.7	435.11
MG	1.5	98.1	1.4	1.5	0.51	6.3	8.0	378.73
ES	1.4	90.3	1.4	1.3	0.51	6.4	8.3	376.50
RJ	1.8	111.3	1.4	1.0	0.52	2.7	9.3	451.75
SP	1.7	99.2	1.2	1.1	0.53	3.0	9.3	533.50
**South ^†^**	1.6	105.3	1.7	1.6	0.47	3.8	8.4	484.11
PR	1.4	102.3	1.7	1.3	0.49	5.2	8.2	455.77
SC	1.4	101.0	1.6	1.8	0.42	2.8	8.6	491.48
RS	1.9	112.6	1.9	1.8	0.49	3.4	8.5	505.08
**Midwest ^†^**	1.4	99.6	1.6	1.2	0.51	5.5	8.6	490.42
MS	1.5	98.2	1.4	1.3	0.48	5.4	8.1	395.50
MT	1.0	98.7	1.7	1.5	0.47	7.1	7.9	380.94
GO	1.3	81.5	1.6	1.4	0.49	6.5	8.1	393.20
DF	1.9	120.0	1.5	0.4	0.60	3.0	10.3	791.99

* Per 100,000 inhabitants; 1 US$ quoted at R $ 3.31 in December 2017; **: coefficient that measures inequality in terms of income distribution in each group, numerically varying from zero to one (the higher the value, the higher the inequality); ^†^ Brazillian regions.

**Table 3:** Number of deaths and mortality standardized by age, genital, and breast cancer according to reproductive period and types of cancer in Brazilian women in 2017.

Type Of Cancer	Total		Reproductive Period		Non-Reproductive Period	
	Deaths	Mortality *	Deaths	Mortality *	Deaths	Mortality *
C50–C57	31,683	35.9	7348	12.6	24,335	91.4
Malignant neoplasm of breast (C50)	16,723	18.9	3761	6.4	12,962	48.6
Malignant neoplasm of cervix uteri (C53)	6385	7.3	2294	3.9	4091	15.5
Malignant neoplasm of corpus uteri (C54)	1827	2.0	135	0.2	1692	6.4
Malignant neoplasm of uterus, part unspecified (C55)	2114	2.4	394	0.7	1720	6.4
Malignant neoplasm of ovary (C56)	3866	4.4	683	1.2	3183	12.0
Malignant neoplasm of vulva (C51) and of vagina (C52)	543	0.6	44	0.1	499	1.8
Malignant neoplasm of other and unspecified female genital organs (C57)	225	0.2	37	0.1	188	0.7

* Standardized by age, for every 100,000 women.

**Table 4:** Factors associated with mortality standardized by age, genital, and breast cancer and reproductive and non-reproductive period in Brazilian resident women in 2017. 95% CI: 95% confidence interval.

Age-Standardized Mortality (per 100,000 Women)	Linear Regression	
	*β* (95%CI)	*p*
**Reproductive Period**		
Primary care coverage (%)	−0.1 (−1.1; 0.9)	0.792
Average years of study *	1.6 (−0.3; 3.6)	0.097
Income per capita **	−0.4(−0.8; −0.03)	0.032
**Non-Reproductive Period**		
Primary care coverage (%)	−0.6(−5.3; 4.0)	0.769
Average years of study *	9.7 (1.5; 18.0)	0.022
Income per capita **	−1.5 (−3.1; 0.1)	0.065

*β*: slope, linear regression; 95% CI: 95% confidence interval; *p* = *p*-value, linear regression; * variation of 1 year of study; ** variation of 100 Brazilian reals (Brazilian currency).
